# Mental disorder trajectories and quality of life among youth residential care leavers: a longitudinal study

**DOI:** 10.1111/jcpp.70210

**Published:** 2026-07-30

**Authors:** Milou Leiting, Katharina Beck, Süheyla Seker, David Bürgin, Delfine d'Huart, Jörg M. Fegert, Nils Jenkel, Cyril Boonmann, Klaus Schmeck, Marc Schmid

**Affiliations:** ^1^ Child and Adolescent Psychiatric Research Department University Psychiatric Clinics Basel, University of Basel Basel Switzerland; ^2^ Department of Social Work Stockholm University Stockholm Sweden; ^3^ Jacobs Center for Productive Youth Development, University of Zurich Zurich Switzerland; ^4^ Department of Clinical Psychology and Translational Psychotherapy Research University of Basel Basel Switzerland; ^5^ Department for Child and Adolescent Psychiatry and Psychotherapy University Hospital Ulm Ulm Germany; ^6^ LUMC Curium—Department of Child and Adolescent Psychiatry Leiden University Medical Center Leiden The Netherlands

**Keywords:** Quality of life, psychopathology, child welfare, out‐of‐home care, children and adolescents, young adults

## Abstract

**Background:**

Adults formerly placed out‐of‐home (care leavers) often accumulate multiple psychosocial adversities that can lead to poor quality of life (QoL) and place them at high risk for developing mental disorders persisting into adulthood. This study examines the development of mental disorders among care leavers and their QoL, differentiating by disorder groups.

**Methods:**

The sample consisted of 119 young adults formerly placed out‐of‐home (*M*_age at baseline_ = 15.0 years; *M*_age at follow‐up_ = 25.4 years). Mental disorders were assessed at baseline using the Kiddie Schedule for Affective Disorder and Schizophrenia—Present and Lifetime Version and at a 10‐year follow‐up with the Structured Clinical Interview for DSM‐5 Disorders—Clinician Version. Personality disorders were evaluated at both time points using the Structured Clinical Interview for DSM‐IV‐TR Axis II. QoL was measured at follow‐up using the World Health Organization QoL‐BREF questionnaire.

**Results:**

Four groups based on the presence or absence of mental disorders at baseline and follow‐up were identified: resilient (*n* = 24, 20.2%), persistent (*n* = 56, 47.0%), remitted (*n* = 25, 21.0%), and newly occurring (*n* = 14, 11.8%). Regarding the mental disorder groups, internalizing disorders at baseline (*β* = −0.44, *p* < .05, 95% CI [−0.85, −0.04]) and internalizing (*β* = −0.78, *p* < .001, 95% CI [−1.14, −0.43]) and externalizing disorders (*β* = −0.50, *p* < .01, 95% CI [−0.86, −0.13]) at follow‐up were negatively associated with QoL in young adulthood.

**Conclusions:**

Mental disorder trajectories from childhood to young adulthood can negatively impact care leavers' QoL. Prevention and early intervention, as well as addressing mental health during out‐of‐home placement and transition into young adulthood, are important to reduce the risk of persistent psychopathology and lower QoL.

## Introduction

Mental disorders frequently emerge during childhood or adolescence (Solmi et al., 2022) and can lead to long‐term consequences for mental health, socioeconomic status, and overall well‐being (Oerlemans, Wardenaar, Raven, Hartman, & Ormel, 2020) and substantially compromise quality of life (QoL) in young adulthood. These risks are particularly pronounced among adults formerly in out‐of‐home care (i.e., care leavers), who are often exposed to cumulative psychosocial adversities and show elevated rates of persistent mental health problems (Cameron et al., 2018; Gypen, Vanderfaeillie, De Maeyer, Belenger, & Van Holen, 2017; Kääriälä & Hiilamo, 2017). After leaving care, these individuals often face ongoing mental health problems and limitations in psychosocial functioning, both of which can impact their well‐being and QoL (Gypen et al., 2017; Leiting et al., 2024). Despite this heightened vulnerability, there is a lack of longitudinal studies on the impacts of mental disorders on QoL in young‐adult care leavers. This study therefore aims to investigate the association between the development of mental disorders from childhood and adolescence into young adulthood and QoL among young‐adult care leavers.

Approximately 12.7% of children in the general population experience mental disorders (Barican et al., 2022). Symptom onset commonly occurs by early adolescence (Solmi et al., 2022). These are linked to long‐term consequences in adulthood such as persistent mental health issues, lower socioeconomic status, social and physical health difficulties (Oerlemans et al., 2020), and reduced QoL, with evidence consistently linking mental disorders to poorer QoL across the general population (Clayborne, Varin, & Colman, 2019).

The risk for mental disorders is particularly elevated for children and adolescents placed in residential care (Beaudry, Yu, Långström, & Fazel, 2021). A meta‐analysis showed that approximately 49% of children and adolescents in residential care meet the criteria for a mental disorder, disruptive behavior disorder (i.e., conduct disorder and oppositional defiant disorder) being the most prevalent (27%), and anxiety and depressive disorders less prevalent (18%) (Bronsard et al., 2016). Furthermore, children and adolescents placed out‐of‐home are often there due to exposures to childhood maltreatment, which significantly increases the risk of mental health problems in this population (Fischer, Dölitzsch, Schmeck, Fegert, & Schmid, 2016). The elevated risk for mental health problems continues into adulthood (McKenna, Donnelly, Onyeka, O'Reilly, & Maguire, 2021). As such, young‐adult care leavers exhibit a significantly higher prevalence of mental disorders (around 30%) compared to the general adult population (Seker et al., 2022). They face an increased risk of suicide compared to their peers who did not receive child welfare services (McKenna et al., 2021) and are at a higher risk of developing substance use disorders (Courtney et al., 2018). Several key factors contribute to the increased risk of mental disorders among care leavers. They include childhood maltreatment, in particular emotional abuse and neglect (Phillips et al., 2024).

QoL, defined by the World Health Organization (WHO) as ‘a person's view of their position in life, considering the cultural and value system they are part of as well as goals, expectations, standards and concerns’ (The Whoqol Group, 1998), is a central indicator to assess overall functioning, particularly in vulnerable populations. Children and adolescents in out‐of‐home care report a lower QoL than their peers (Larsen, Goemans, Baste, Wilderjans, & Lehmann, 2021). Maltreatment and abuse experiences are common among children placed in out‐of‐home care, and they have been negatively associated with QoL in adolescents living in residential care (Greger, Myhre, Lydersen, & Jozefiak, 2016). Other factors that affected QoL in a Swiss cohort of children and adolescents in child welfare and juvenile justice institutions were reduced social participation, mental health problems, and separation from their biological families, while symptom reduction improved QoL (Gander et al., 2019). Although research on QoL in young‐adult care leavers is limited, the available studies have reported reduced life satisfaction and adverse adult outcomes (Cameron et al., 2018; Kääriälä & Hiilamo, 2017; Sariaslan et al., 2022). Studies from Nordic countries have pointed to poorer outcomes regarding self‐support (i.e., receipt of social assistance, unemployment, and lower income), education, mental health, criminality, early parenthood, mortality, suicidal behavior, substance abuse, and disability pension receipt (Kääriälä & Hiilamo, 2017). Similar patterns have been observed across England and Germany, where care leavers experience faster transitions into adulthood, higher risks for unemployment, lower postsecondary educational attainment, and often a lack of social support (Cameron et al., 2018). They also face elevated risks of homelessness and involvement with the justice system (Gypen et al., 2017).

In summary, mental disorders beginning in childhood often persist into adulthood, lowering QoL and leading to adverse adult outcomes. Young people in out‐of‐home care are particularly vulnerable and continue to face health, economic, and social challenges after leaving care. However, no study has examined the effect of the development of mental disorders from childhood and adolescence into young adulthood on QoL in young‐adult care leavers. Addressing this gap could offer valuable knowledge for policymakers on why and how to better support youths transitioning out of care. The aim of this study is therefore to investigate the relationship between mental disorder trajectories and QoL in young‐adult care leavers. Specifically, it explores (a) how the remission (any mental disorder only at baseline), persistence (any mental disorder at both time points), late onset (any mental disorder only at follow‐up), or absence of mental disorders (no disorder at either time point) influence QoL, (b) whether different mental disorders experienced during residential care have a differential impact on QoL after youths leave care, and (c) how current mental health disorders shape care leavers' QoL.

## Methods

### Study design and procedure

This study includes participants from the project Swiss Study for Clarification and Goal‐Attainment in Youth Welfare and Juvenile Justice Institutions (German: Modellversuch Abklärung und Zielerreichung in stationären Massnahmen; MAZ.; Schmid, Kölch, Fegert, & Schmeck, 2013) and its follow‐up project Youth Welfare Trajectories: Learning from Experience (German: Jugendhilfeverläufe: Aus Erfahrung lernen; JAEL; Jenkel et al., 2025). The MAZ. baseline project (2007–2011) surveyed 592 children and adolescents from 64 Swiss youth welfare and juvenile justice institutions accredited by the Federal Office of Justice. The baseline study only involved child welfare and juvenile justice institutions accredited by the Swiss Federal Office of Justice. This accreditation refers to a formal regulatory process requiring institutions to meet defined legal, structural, and quality standards (e.g., professional qualifications of staff, provision of 24‐hr care, and pedagogical concepts), and includes regular monitoring. Institutional types reflect the diverse and eclectic landscape of the Swiss integrated civil‐penal structure. Types include residential youth care, group homes, boarding schools, special education schools, transitional programs, and multidisciplinary assessment stations. Most operate as open settings, though some include locked wards for youth offenders. Within these institutions, only youths who had been placed in an institution for at least 1 month were eligible to participate. Exclusion criteria for the initial enrollment in the study included severe cognitive impairment that would prevent participation and severely reduced linguistic competence, which would make an interview with the person concerned impossible. It is important to note that, in Switzerland, out‐of‐home placements are organized within a publicly regulated system involving both child welfare and juvenile justice authorities. Children and adolescents are placed out of home either for child protection reasons (e.g., maltreatment, neglect, or family dysfunction), following judicial measures related to offending behavior, or due to other reasons. Importantly, these placements are not strictly separated by jurisdiction into distinct institutional types. Thus, youths with placements due to civil or penal law may reside in the same residential care institutions, with placement decisions being primarily guided by their individual treatment and educational needs (Jäggi et al., 2021).

During the follow‐up project, JAEL (2018–2022; mean follow‐up time = 9.7 years), the same youths, who were by then young adults, were contacted again, and 231 participants were assessed by trained psychologists using psychometric questionnaires, semi‐structured clinical interviews, and qualitative interviews, corresponding to a follow‐up response rate of 45.2% among participants who had agreed to be recontacted (231/511). Study participants were included in JAEL if they (1) had previously taken part in the baseline MAZ. project and (2) had consented to being contacted for any follow‐up project. For comprehensive information on the research project, consult other studies published with this sample (d'Huart et al., 2022; Seker et al., 2022). The JAEL study was approved by the Ethics Committee of Northwestern and Central Switzerland (EKNZ, Ref.: 2017‐00718). The reporting of the present study follows the Strengthening the Reporting of Observational Studies in Epidemiology (STROBE) checklist (Table S1).

### Measurements

#### Mental disorders

At baseline (MAZ.), mental disorders were assessed using the Kiddie Schedule for Affective Disorder and Schizophrenia—Present and Lifetime Version (K‐SADS‐PL) (Kaufman et al., 1997), a standardized semi‐structured interview designed for diagnosing current and past mental disorders in children and adolescents aged 6–18 years according to the Diagnostic and Statistical Manual of Mental Disorders—Fourth Edition (DSM‐IV). Items were rated on a 4‐point Likert scale (0 = *no information available*, 1 = *not present*, 2 = *subthreshold level*, 3 = *threshold level*). Personality disorders in childhood and adolescence were assessed with the Structured Clinical Interview for DSM‐IV‐TR Axis II Personality Disorders (SCID‐II) (First, Spitzer, Gibbon, & Williams, 1995), a semi‐structured diagnostic interview with robust interrater reliability (Maffei et al., 1997) that evaluates 10 different personality disorders, with items scored on a 3‐point Likert scale (1 = *absent*, 2 = *subthreshold*, 3 = *threshold*). Both interviews were administered at the same assessment point (MAZ.) during residential placement. For this study, we included only current diagnoses present at the time of the out‐of‐home placement.

At follow‐up (JAEL), mental disorders were assessed using the Structured Clinical Interview for DSM‐5 Disorders—Clinician Version (SCID‐5‐CV) (First, Williams, Karg, & Spitzer, 2016), a semi‐structured diagnostic tool for DSM‐5 diagnoses with dichotomous item format (0 = *not present*, 1 = *present*). The SCID‐5‐CV has demonstrated high reliability and clinical validity (Osório et al., 2019). Personality disorders at follow‐up were again assessed with the SCID‐II (First et al., 1995).

All mental disorders were coded according to the *International Classification of Diseases 10th Revision* (ICD‐10) F‐codes and grouped following the Hierarchical Taxonomy of Psychopathology (HiTOP) framework (Kotov et al., 2022), to ensure comparability of disorders diagnosed according to the DSM‐IV and DSM‐5, respectively (Table S2). HiTOP organizes psychopathology dimensionally and hierarchically based on symptom covariation; subfactors (sexual problems, eating pathology, fear, distress, mania, substance abuse, antisocial behavior) are grouped into spectra (internalizing, externalizing, thought disorder, detachment, externalizing), and these spectra combine at the top level into a general *p* factor (see Appendix S1).

Lastly, participants were classified into four mental disorder trajectories based on the presence or absence of any diagnosis at baseline and follow‐up: persistent, (any mental disorder at both time points), remitted (only at baseline), newly occurring (only at follow‐up), and resilient (no disorder at either time point). This categorization follows previous work published with this cohort (Seker, Boonmann, d'Huart, et al., 2022). Further details on the ICD‐10 classification of the data from the JAEL project within the HiTOP framework can be found in the 2022 study by Seker, Boonmann, d'Huart, et al. (2022).

#### Quality of life (QoL)

At follow‐up (JAEL), QoL was assessed using the WHO Quality‐of‐Life questionnaire (WHOQoL‐BREF), which was developed in 1998 (The Whoqol Group, 1998). It is a 26‐item self‐report questionnaire that assesses QoL in four domains (psychological, social, physical, environmental) as well as overall QoL. The rating of each item is based on a 5‐point Likert scale ranging from 1 (*not at all*) to 5 (*completely*), resulting in a total score between 26 and 130. For our analyses, we used the four QoL domain scores and the overall QoL score. Internal consistency revealed a Cronbach's *α* = .78 for psychological QoL, *α* = .82 for physical QoL, *α* = .80 for environmental QoL, *α* = .74 for social QoL and *α* = .93 for total QoL. The moderate‐to‐large Cronbach's alpha values were consistent with the psychometric evaluation in the original study.(Skevington, Lotfy, & O'Connell, 2004) The population means for German young adults are 84.11 (*SD* = 13.98) for the physical domain, 77.62 (*SD* = 14.95) for the psychological, 74.83 (*SD* = 18.61) for the social, and 71.47 (*SD* = 13.54) for the environmental (Angermeyer, Kilian, & Matschinger, 2000).

### Statistical analysis

Missing data were present in 37.7% of the total sample (*N* = 231), namely, 10.8% from the WHOQOL‐BREF, 12.1% from the SCID‐II and SCID‐5‐CV, and 14.7% from the K‐SADS‐PL. Missing data were handled using complete‐case analysis. Imputation was not performed because the primary variables of interest were clinical diagnoses assessed at two time points, and imputing such categorical diagnostic data may introduce misclassification and generate implausible patterns of mental health status over time. Comparisons between included and excluded participants did not reveal systematic differences in key sociodemographic variables, except for a small difference in age (see Table S3). Given the small magnitude of this difference, there was no indication of substantial selection bias.

Absolute and relative frequencies of sociodemographic characteristics of the sample were calculated. Prevalence rates for the four trajectory groups were calculated with descriptive statistics. To assess differences in QoL (psychological, social, physical, environmental, and overall) across groups, we conducted five separate analyses of variance (ANOVA), followed by Tukey's honestly significant difference test (HSD) test for post hoc comparisons, which corrected for multiple comparisons within each ANOVA. Lastly, we examined the associations between the presence or absence of any disorder from each specific HiTOP spectrum (i.e., internalizing, thought, detachment, and externalizing disorders, respectively), at baseline and follow‐up, respectively, and the WHOQoL‐BREF total score at follow‐up through standardized regression analyses, adjusting for gender and age by using a Wald *t*‐distribution approximation. All analyses were conducted using R (version 4.3.3) (R Core Team, 2024). All results were considered significant if the 95% confidence interval excluded 0 and *p* values were *p* < .05.

## Results

### Sociodemographic characteristics

Out of the 592 participants in the research project MAZ., 511 had agreed to be contacted again for a follow‐up study. After significant efforts, the study team included 231 participants in the JAEL project (Figure 1). Participants with missing data in the questionnaires of interest were excluded from this study (*n* = 87), as were participants over the age of 18 at baseline (MAZ., *n* = 25). The total sample consisted of 119 formerly out‐of‐home placed young adults (*M*_age at baseline_ = 15.0 years, *SD* = 2.2, range = 7.3–17; *M*_age at follow‐up_ = 25.4, *SD* = 2.4, range = 17.1–30.5), 35.3% of whom were female. The response rate of the original sample was 21.0% (Table 1). Included and excluded participants differed only in age: included participants were significantly younger by approximately 1 year, *t*(254) = −4.1, *p* < .001. All other key sociodemographic characteristics did not show any statistically significant differences (Table S3).

**Figure 1:**
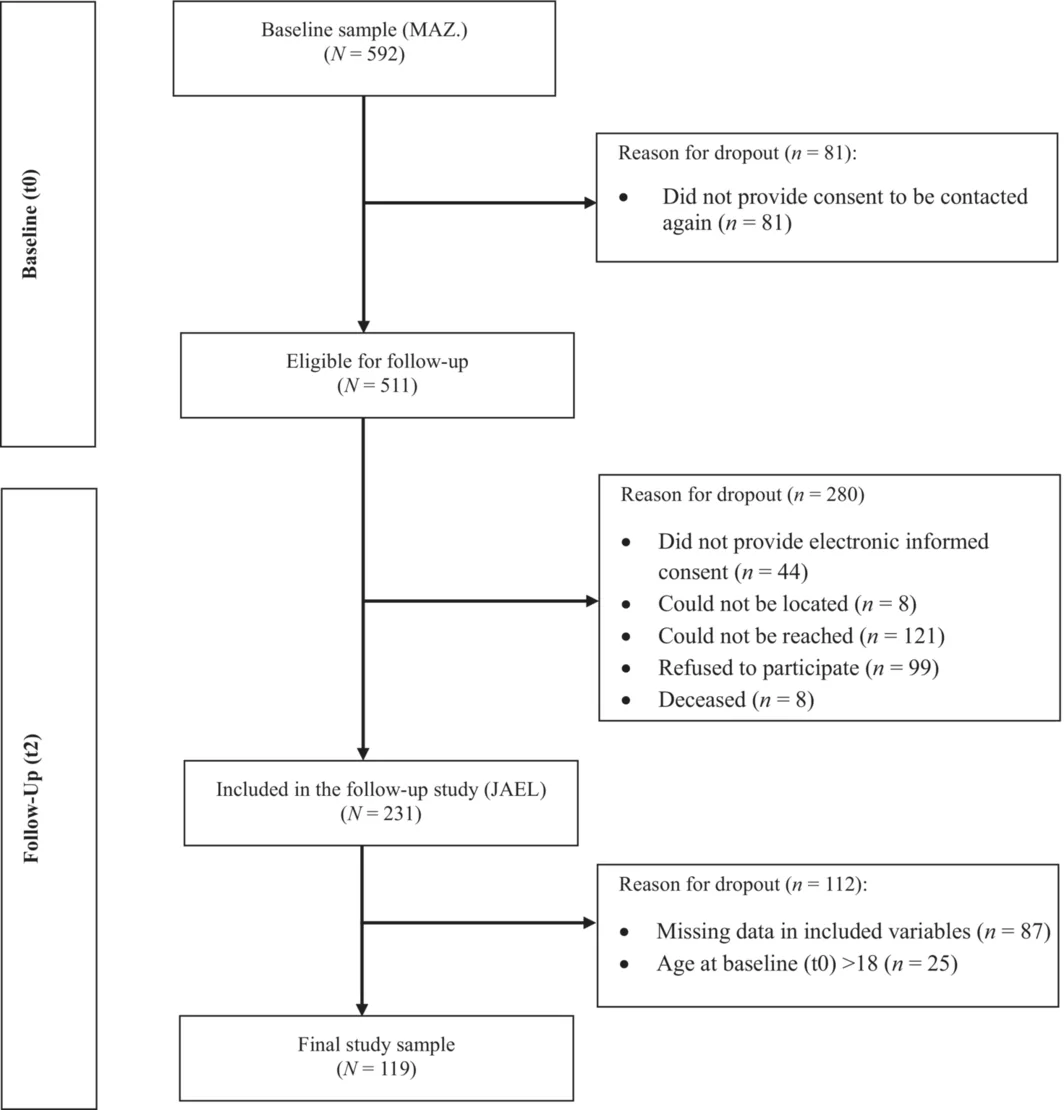
Flowchart of study participants

**Table 1:** Sociodemographic characteristics of the study sample (*N* = 119).

Characteristics	*M* [*SD*] (range)/*n* (%)
Age at baseline (years)	15.0 [2.2] (7.3–17.0)
Age at follow‐up (years)	25.4 [2.4] (17.1–30.5)
Women	42 (35.3)
Swiss nationality	94 (79.0)
Reason for placement (baseline)	
Child welfare	65 (54.6)
Juvenile justice	19 (16.0)
Other	34 (28.6)
In a partnership	75 (63.0)
Education	
Higher education	9 (7.6)
Secondary education	9 (7.6)
Apprenticeship	60 (50.4)
Mandatory school	38 (31.9)
Did not complete mandatory school	3 (2.5)
Currently employed	68 (57.1)
Social welfare support	32 (26.9)
Current psychotherapeutic treatment	26 (21.9)
Time between baseline and follow‐up assessments (years)	10.3 [1.0] (8.4–12.6)
Placement characteristics	
Number of previous placements (baseline)	0.63 [1.1] (0–5)
Number of total placements (follow‐up)	3.5 [3.4] (1–20)
Reasons for leaving residential care	
Planned transition^a^	84 (73.7)
Unstable circumstances^b^	30 (26.3)
Timing and duration of placements	
Age at first placement (years)	11.2 [4.6] (0–18)
Time in residential care before baseline assessment (years)	0.90 [0.80] (0–3.3)
Approximate time between beginning of first and end of last placement (years)	6.5 [4.6] (0–17)
Mental disorder at baseline (MAZ)	
Internalizing disorders	35 (29.4)
Detachment disorders	6 (5.0)
Thought disorders	4 (3.4)
Externalizing disorders	62 (52.1)
Any mental disorder	81 (68.1)
Mental disorder at follow‐up (JAEL)	
Internalizing disorders	40 (33.6)
Detachment disorders	11 (9.2)
Thought disorders	11 (9.2)
Externalizing disorders	58 (48.7)
Any mental disorder	70 (58.8)

More information on the living situation after leaving care is available in Table S7. *M*, mean; *N*, number of participants; *SD*, standard deviation.

^a^ Regular exit, age‐related exit.

^b^ For example, rule violations, running away, or termination by caregivers.

### Mental disorder trajectory groups and QoL

First, our results indicated that the most prevalent trajectory group was persistent (*n* = 56, 47%), followed by remitted (*n* = 25, 21%). The resilient trajectory group was the third largest, with 24 participants (20.2%) who did not meet the criteria for a mental disorder at baseline or follow‐up. Newly occurring was the least prevalent trajectory group, with 14 participants (11.8%) who met the criteria for a mental disorder only at follow‐up. Overall, 68.1% of the participants (*n* = 81) exhibited a mental disorder at baseline, and 58.8% (*n* = 70) at follow‐up (Figure S1).

Second, we tested for statistically significant differences in QoL between the four trajectory groups. Figure 2 displays the mean QoL scores for all domains grouped by trajectory. Results indicated significant differences in overall QoL: *F*(3, 115) = 4.13, *p* = .008. A post hoc Tukey's HSD test revealed that the resilient group (*M* = 76.3, *SD* = 13.3) had significantly higher overall QoL compared to the newly occurring group (*M* = 61.1, *SD* = 19.6; *p* = .017) and the persistent group (*M* = 65.5, *SD* = 19.6; *p* = .020) (Figure 2A). The second ANOVA evaluating physical QoL showed significant differences: *F*(3, 115) = 5.27, *p* < .01. The resilient group (*M* = 82.7, *SD* = 12.7) had significantly higher physical QoL compared to the newly occurring group (*M* = 66.8, *SD* = 17.6; *p* = .023) and the persistent group (*M* = 69.1, *SD* = 19.2; *p* = .005) (Figure 2B). The third ANOVA indicated significant differences regarding psychological QoL: *F*(3, 115) = 3.54, *p* = .017. A post hoc test revealed that the resilient group (*M* = 71.5, *SD* = 16.5) had significantly higher psychological QoL compared to the newly occurring group (*M* = 53.9, *SD* = 25.8; *p* = .040) (Figure 2C). The ANOVAs for the environmental (Figure 2D) and social QoL (Figure 2E) domains did not reveal any statistically significant differences. However, the mean observed differences followed the same pattern as in the other QoL domains; the resilient group consistently showed the highest QoL, followed by the remitted group, while the persistent and newly occurring group had the lowest QoL scores. Population norms for each domain are indicated by the gray line (for more information, see Table S4).

**Figure 2:**
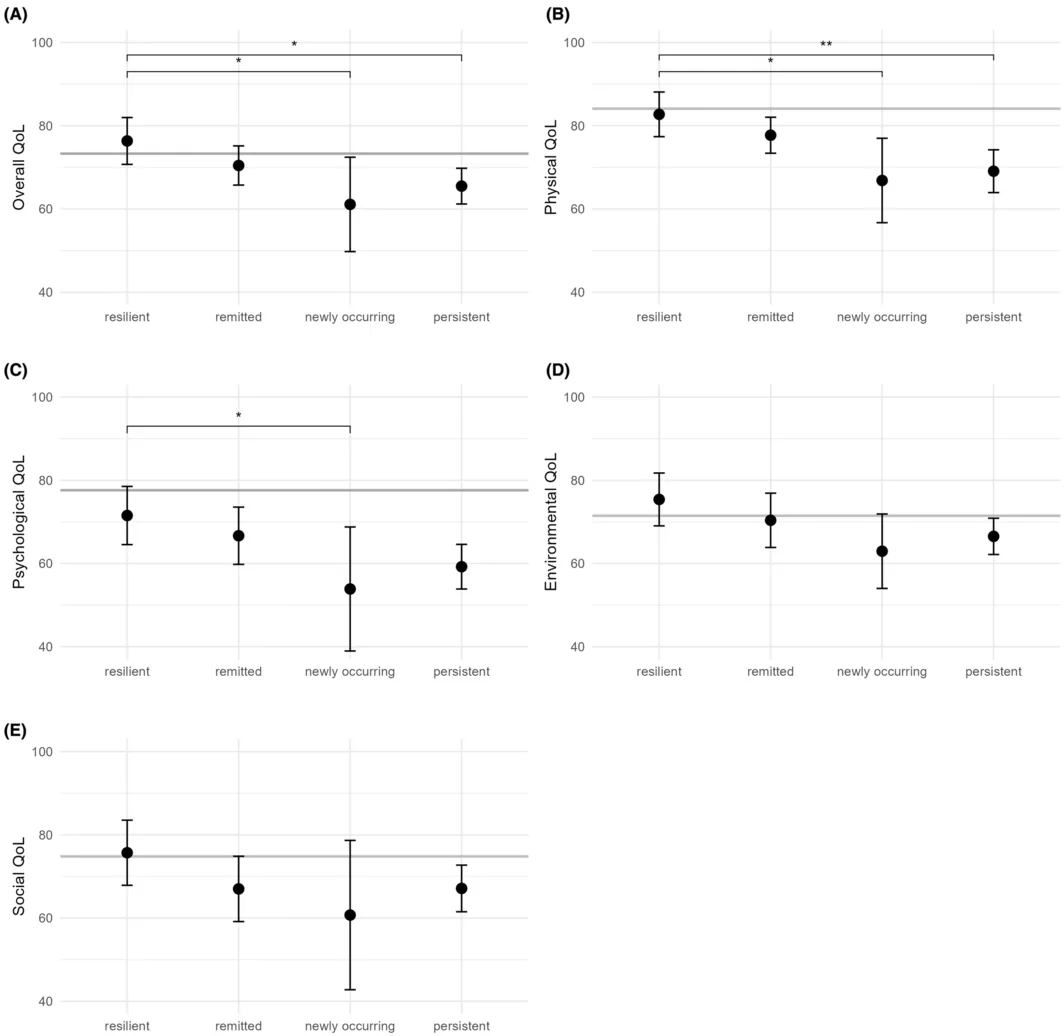
Mean quality of life scores for each domain and mental disorder trajectory (A) Overall QoL. (B) Physical QoL. (C) Psychological QoL. (D) Environmental QoL. (E) Social QoL. Gray horizontal lines indicate the mean of the norm population (Angermeyer et al., 2000) for the respective QoL domain. Sample sizes for mental disorder trajectories are the following: *n*_persistent_ = 56; *n*_remitted_ = 25; *n*_resilient_ = 24; *n*_newly occurring_ = 14. Error bars represent 95% confidence intervals. **p < .01, *p < .05.

### Impact of adolescent mental disorders on overall QoL in young adulthood

The results of the multiple regression analyses, which were adjusted for gender and age, on the associations between mental disorders (as classified based on the HiTOP framework) during childhood and adolescence and QoL in young adulthood are reported in Table 2.

**Table 2:** Associations between adolescent mental disorders and overall quality of life in young adulthood

Dependent variable	Independent variable	Stand. Beta	*SE*	*p* Value	95% CI	*f* ^2^
Overall QoL	Any mental disorder	−0.22	0.195	ns	−0.60, 0.17	0.050
	Gender	−0.42	0.194	**<.05**	−0.80, −0.04	
	Age	0.05	0.093	ns	−0.14, 0.23	
Overall QoL	Externalizing disorders	−0.30	0.184	ns	−0.30, 0.43	0.041
	Gender	−0.81	0.195	**<.05**	−0.81, −0.04	
	Age	−0.15	0.095	ns	−0.15, 0.23	
Overall QoL	Internalizing disorders	−0.44	0.207	**<.05**	−0.85, −0.04	0.083
	Gender	−0.29	0.201	ns	−0.69, 0.11	
	Age	0.02	0.092	ns	−0.17, 0.20	
Overall QoL	Detachment disorders	−0.004	0.408	ns	−0.81, 0.80	0.060
	Gender	−0.43	0.195	**<.05**	−0.81, −0.04	
	Age	0.22	0.126	ns	−0.04. 0.46	
Overall QoL	Thought disorders	−0.64	0.514	ns	−1.66, 0.34	0.053
	Gender	−0.47	0.196	**<.05**	−0.86, −0.08	
	Age	0.06	0.094	ns	−0.12, 0.25	

Mental disorders were assessed with the Kiddie Schedule for Affective Disorder and Schizophrenia—Present and Lifetime Version (K‐SADS‐PL) and the Structured Clinical Interview for DSM‐IV‐TR Axis II Personality Disorders (SCID‐II) and classified based on the HiTOP spectrum categories. CI, confidence interval; ns, not significant (*p* > .05); *SE*, standard error; *f* ^2^ = effect size (values of 0.02, 0.15, and 0.35 indicate small, medium, and large effects, respectively). See Table S8 for additional results on the associations between adolescent mental disorders and overall quality of life in young adulthood, adjusted for psychotherapeutic treatment. Significant values are presented in bold.

The association between mental disorders and overall QoL was consistently negative, but significant only in the case of internalizing disorders. More specifically, the effect of internalizing disorders on QoL in young adulthood was negative and statistically significant (*β* = −0.44, 95% CI [−0.85, −0.04], *p* = .03), and the coefficient of determination (*R* ^2^) indicated a proportion of 7.8% of explained variance by the independent variables. All other regression models did not reach significance, as shown by both the *p* values and the 95% confidence intervals. Nonetheless, the standardized beta coefficients for the nonsignificant regression models ranged from a rather small coefficient (*β* = −0.004) to a higher coefficient (*β* = −0.64), suggesting a potentially meaningful negative association despite not reaching the significance threshold.

Last, participants who had at least one diagnosis at baseline did not report significantly lower total QoL at follow‐up (*M* = 66.6, *SD* = 14.9) compared to those without a diagnosis: *M* = 70.7, *SD* = 16.4; *F*(1, 108) = 1.68, *p* = ns. Correlation coefficients between mental disorders at baseline and the QoL domain can be found in Table S5.

### Impact of current mental disorders on current overall QoL

The results of the multiple regression analyses, which were adjusted for gender and age, on the associations between currently present mental disorders (as classified based on the HiTOP framework) and QoL in young adulthood are reported in Table 3.

**Table 3:** Associations between current mental disorders and current overall quality of life

Dependent variable	Independent variable	Stand. Beta	*SE*	*p* Value	95% CI	*f* ^2^
Overall QoL	Any mental disorder	−0.56	0.177	**<.01**	−0.91, −0.21	0.136
	Gender	−0.42	0.184	**<.05**	−0.79, −0.06	
	Age	0.08	0.088	ns	−0.09, 0.26	
Overall QoL	Externalizing disorders	−0.50	0.185	**<.01**	−0.86, −0.13	0.110
	Gender	−0.51	0.188	**<.01**	−0.88, −0.14	
	Age	0.14	0.093	ns	−0.05, 0.32	
Overall QoL	Internalizing disorders	−0.78	0.180	**<.001**	−1.14, −0.43	0.217
	Gender	−0.31	0.179	ns	−0.67, 0.04	
	Age	0.07	0.085	ns	−0.10, 0.24	
Overall QoL	Detachment disorders	−0.57	0.310	ns	−1.19, 0.04	0.076
	Gender	−0.42	0.189	ns	−0.79, 0.05	
	Age	0.06	0.091	ns	−0.12, 0.25	
Overall QoL	Thought disorders	−0.51	0.310	ns	−1.13, 0.10	0.070
	Gender	−0.42	0.189	<.05	−0.80, −0.05	
	Age	0.07	0.091	ns	−0.11, 0.25	

Mental disorders were assessed with the Structured Clinical Interview for DSM‐5 Disorders—Clinician Version (SCID‐5) and the Structured Clinical Interview for DSM‐IV‐TR Axis II Personality Disorders (SCID‐II) and classified based on the HiTOP spectrum categories. CI, confidence interval; ns, not significant (*p* > .05); *SE*, standard error; *f* ^2^ = effect size (values of 0.02, 0.15, and 0.35 indicate small, medium, and large effects, respectively). Significant values are presented in bold.

The association between mental disorders at follow‐up and overall QoL at follow‐up was consistently negative and significant in the case of internalizing and externalizing disorders. More specifically, the impact of the presence of any internalizing disorder on overall QoL was negative and statistically significant (*β* = −0.78, 95% CI [−1.14, −0.43], *p* < .001), and *R* ^2^ indicated a proportion of 17.8% of explained variance by the independent variables. Next, the presence of any externalizing disorder was significantly negatively correlated with overall QoL (*β* = −0.50, 95% CI [−0.86, −0.13], *p* < .01). The presence of any externalizing disorder explained 10% of the variance in overall QoL. Last, the presence of any mental disorder was negatively and significantly associated with overall QoL (*β* = −0.56, 95% CI [−0.91, −0.21], *p* < .01), and 12% of the variance in QoL was explained by the presence of any mental disorder. Notably, detachment and thought disorders showed a rather large beta coefficient, −0.57 and −0.51, respectively. Last, participants with more than one diagnosis reported significantly lower overall QoL (*M* = 64.8, *SD* = 17.0) compared to those without a diagnosis: *M* = 73.6, *SD* = 12.8, *F*(1, 112) = 8.78, *p* < .01. Results of the correlations between the presence or absence of any mental disorder at follow‐up and each QoL domain can be found in (Table S6).

## Discussion

The present study explored how mental disorders from childhood and adolescence into adulthood relate to QoL in formerly out‐of‐home placed young adults. Nearly half of the sample followed a persistent trajectory, fulfilling the criteria for a mental disorder both at baseline and at follow‐up. These participants, along with those who had a disorder at follow‐up only (i.e., the newly occurring group), reported significantly lower overall, physical, and psychological QoL than participants without a disorder at either time point. Internalizing disorders in childhood and adolescence increased the likelihood of a lower QoL in young adulthood, while externalizing and internalizing disorders in adulthood were both associated with lower QoL.

The outcome that most youth residential care leavers showed persistent mental disorders from adolescence to adulthood was in line with prior studies (Beaudry et al., 2021; Bronsard et al., 2016; Bürgin et al., 2023). At baseline, almost 70% met criteria for a mental disorder, and approximately 60% still did so at follow‐up, which means that most still struggled in terms of mental health (d'Huart et al., 2022; Gypen et al., 2017; McKenna et al., 2021; Seker, Boonmann, d'Huart, et al., 2022). A meta‐analytic review reported that 30% of young‐adult care leavers experience at least one mental disorder, higher than the general population (Seker, Boonmann, d'Huart, et al., 2022), but lower than in our sample. The lower prevalence in that meta‐analysis may reflect methodological differences across studies, that is, reliance on self‐report measures, the inclusion of foster care participants, and the exclusion of personality disorders. Moreover, in the present study, both persistent and newly occurring trajectories showed a pattern of lower QoL than the resilient group, which aligns with prior research showing that cumulative early exposure to mental disorders can predict greater adverse outcomes in adulthood (Copeland, Wolke, Shanahan, & Costello, 2015) and is associated with reduced QoL (Hohls, König, Quirke, & Hajek, 2021). One possible explanation for this pattern could be that the transition into independent living represents an often destabilizing and vulnerable period for care leavers. The increased stress associated with this transition may intensify existing psychopathology. In addition, continuity of mental health care is not always ensured during this period, which may contribute to symptom persistence, re escalation, or more chronic trajectories (Baidawi, Mendes, & Snow, 2014). At the same time, transitions out of care were heterogeneous in our sample. While most participants experienced planned exits (e.g., age‐related discharge), a smaller proportion left under more unstable circumstances (e.g., rule violations or unplanned termination) (Table 1). These differing pathways may have implications for later adjustment and QoL, although this was not explicitly examined in the present study.

Unsurprisingly, participants who did not experience a mental disorder in adolescence and young adulthood showed higher QoL scores. This supports evidence of higher QoL for individuals who never had mental disorders (Schechter, Endicott, & Nee, 2007). As highlighted by previous research, key resilience factors (such as self‐efficacy, problem‐solving, and self‐regulating abilities) could help children and adolescents placed out‐of‐home cope with challenges (Lou, Taylor, & Di Folco, 2018). Finally, although this difference did not reach statistical significance, participants who only experienced a mental disorder in adolescence showed a tendency towards higher QoL scores compared to the persistent and newly occurring groups, which could suggest that remission from a mental disorder can be associated with higher QoL (Bastiaansen, Ferdinand, & Koot, 2020). However, even when they do not fulfill the criteria for any mental disorder in adulthood, individuals with a mental disorder in adolescence could continue to experience mental health issues at subclinical level, and such issues could lead to daily impairments. This aligns with prior evidence suggesting that a complete restoration of QoL after remission is relatively rare (Hohls et al., 2021). To sum up, newly occurring and persistent trajectory groups had a QoL below population norms, which underscores how young‐adult care leavers are an at‐risk population in need of targeted support (Akister, Owens, & Goodyer, 2010; Cameron et al., 2018; Gypen et al., 2017; Kääriälä & Hiilamo, 2017). In contrast to the general population, where remission of mental disorders is more frequently observed (Caspi & Moffitt, 2018), the remitted group in our sample was small, and the persistent group was the largest, which highlights the high mental health burden in this population. In this high‐risk population, remission is likely influenced by multiple factors, including therapeutic interventions, changes in environmental conditions (e.g., removal from adverse family environments, access to supportive relationships), and individual resilience processes. Protective factors such as social support (Leiting et al., 2025), higher self‐efficacy and self‐directedness (Leiting et al., 2024) may be particularly relevant in this context. The resilient and remitted groups in our study, though smaller, exhibited QoL scores comparable to population norms, indicating that some care leavers can adapt well despite significant risk factors.

We also analyzed how our results were associated with the different HiTOP spectra. Internalizing disorders in childhood and adolescence were associated with lower QoL in young adulthood, which aligns with previous research linking adolescent mental disorders to poorer adult physical health and social relationships and to an overall lower QoL (Clayborne et al., 2019). By contrast, no significant associations emerged between adolescent externalizing, detachment, thought disorders in childhood and adolescence, and adult QoL. This is inconsistent with findings suggesting that disorders from these groups, which include both personality disorders and schizophrenia spectrum disorders, are associated with adverse adult outcomes (Skodol, Johnson, Cohen, Sneed, & Crawford, 2007) and reduced QoL (Chen et al., 2006). A likely explanation is the small number of participants who reported detachment and thought disorders, which limited the statistical power and may have obscured significant associations. Additionally, a recent systematic review highlighted that symptom‐based approaches could reveal stronger associations with adult mental health outcomes than dichotomizing psychopathology, which excludes subclinical symptoms (Mulraney et al., 2021).

Adult internalizing and externalizing disorders were both associated with reduced QoL in young adulthood. These results are well‐aligned with prior work showing that current mental disorders are associated with poorer QoL in adults (Evans, Banerjee, Leese, & Huxley, 2007). A further study reported that current mental disorders, especially internalizing disorders, are strongly associated with a substantial loss of QoL (The ESEMeD/MHEDEA 2000 investigators et al., 2004). Finally, no significant associations were found between adult thought and detachment disorders and QoL, which is inconsistent with previous studies on clinical populations (Ridgewell, Blackford, McHugo, & Heckers, 2017). This could be due to the small number of participants (*n*_detachment,thought_ = 11) reporting a mental disorder that falls into these disorder groups in our sample (d'Huart et al., 2022). However, the association between current detachment disorders and QoL was close to reaching statistical significance, suggesting that a larger sample might exhibit significant effects.

### Limitations and strengths

This study has several limitations. First, the sample size (*N* = 119) was relatively small, and the retention rate was only moderate at 21.0%, though comparable to other similar studies. Attrition analyses did not reveal a systematic dropout pattern (Table S3). The only difference we found between included and excluded participants was in their age, which may have been due to the exclusion of participants over the age of 18 at baseline. For that reason, age was a control variable in our analyses. Second, the number of participants in each of the four trajectory groups was limited, which could have diminished the statistical significance. Third, mental health was assessed at only two time points (during residential care and approximately 10 years later, after the participant had left care), so recovery or critical periods between the measurements may have been missed. Accordingly, the categorization into groups represents simplified patterns of diagnostic status rather than true longitudinal trajectories or courses of psychopathology, and does not allow conclusions about symptom continuity or fluctuations between assessments. Still, it is important to acknowledge that young‐adult care leavers constitute a hard‐to‐reach and often highly vulnerable population, and multiple assessments over time may have posed significant challenges for them. Fourth, mental disorders were grouped according to the HiTOP framework, which slightly differs from more commonly used categorical classifications of internalizing and externalizing disorders. While this approach allows for a more dimensional representation of psychopathology, it may limit comparability with studies using traditional diagnostic groupings. Fifth, mental disorders were assessed using different diagnostic systems and instruments across time (DSM‐IV‐based K‐SADS‐PL at baseline and DSM‐5‐based SCID‐5 at follow‐up). Although diagnoses were harmonized using ICD‐10 classifications and aggregated into broader categories, differences between diagnostic frameworks and assessment instruments may have introduced some degree of measurement inconsistency across time points. Lastly, our sample may not be fully representative of care leavers in other countries, as Switzerland's youth welfare system and policies have unique characteristics (Jäggi et al., 2021). Nonetheless, our results are broadly consistent with international research on this population.

Despite these limitations, our study has notable strengths. First, to the best of our knowledge, this is the first study to use QoL measures to investigate the lives of care leavers, either with or without mental disorders. Second, using validated semi‐structured clinical interviews to assess mental disorders at both baseline and follow‐up is methodologically strong and ensured reliable assessments of mental disorders at both time points. Third, the thorough assessment of mental disorders, both at baseline and after a 10‐year follow‐up, within a longitudinal cohort of young‐adult residential care leavers is a notable strength. This is a particularly hard‐to‐reach population and to date no other research project has longitudinally followed Swiss young‐adult care leavers using standardized interviews (d'Huart et al., 2022; Seker, Boonmann, d'Huart, et al., 2022).

### Future research and implications

More research on the QoL of young‐adult care leavers is needed, particularly on their transition into independent adult life. Longitudinal assessments from childhood onward, ideally across multiple time points, could offer deeper insights into the complex relationship between mental health and QoL across their lifespans. Additionally, as these findings are of an exploratory nature, replicating them in larger samples or other residential care contexts and investigating other adult outcomes influenced by mental disorders such as educational and occupational outcomes could further highlight the ongoing support needs of this population.

Our findings call for practical recommendations. Addressing mental disorders in residential care and after leaving care is indispensable. During residential care, evidence‐based treatments should be implemented, as they have been proven to be more effective than usual institutional care (De Swart et al., 2012), while a therapeutic milieu approach can further enhance functional outcomes (Huefner & Ainsworth, 2021). It is important to underline that mental disorders themselves, beyond the experience of residential care, are crucial for the QoL of care leavers. For that reason, delivering effective treatments during out‐of‐home placement is important, as this will likely improve long‐term outcomes such as QoL, prevent persistent symptoms, and increase the chances that young care leavers will be able to cope successfully with the challenges they face in life. Diagnoses of mental disorders in childhood and adolescence alone are not a determinant of future QoL outcomes. Instead, monitoring the progression of mental health, also on a subclinical basis, may be a more effective way to support care leavers.

Last, a continuity of support during the transition into a more independent adult life, but also during the transition from child and adolescent psychiatric settings to adult psychiatry, should be a priority to enhance mental health outcomes and QoL. At the same time, interventions should focus on identifying and fostering individual resilience factors in youths in residential care.

## Conclusions

This study investigated how mental disorder trajectories from childhood and adolescence to young adulthood relate to QoL in young‐adult care leavers. Persistent and newly developed mental disorders were linked to reduced QoL in young adulthood, while those without mental disorders showed higher QoL. Childhood and adolescent internalizing disorders increased the risk of poor QoL, and both adult externalizing and internalizing disorders were associated with lower adult QoL. These findings highlight the importance of preventing and treating mental disorders during out‐of‐home placement to avoid chronic mental disorders and foster healthy and functional life outcomes for care leavers. Importantly, being placed outside the biological family does not inevitably lead to a poorer QoL; some care leavers show resilient trajectories with a high QoL. However, those with ongoing mental health issues remain at greater risk and face greater challenges in life. It is therefore essential—especially during the transitional period into independent adult life—to provide ongoing psychological treatments and to foster protective factors for youth residential care leavers in order to increase their functionality and QoL in adulthood.

## Ethical considerations

Informed consent for participation in the study was obtained from all participants, or their legal representatives when required. The JAEL study was approved by the Ethics Committee of Northwestern and Central Switzerland (EKNZ; approval date: June 2017; reference number: 2017‐00718).

**Key points**

**What's known?**

- Mental disorders often begin in childhood or adolescence and can have long‐term consequences for the well‐being of young adults.
- Adults formerly placed out‐of‐home (care leavers) accumulate multiple psychosocial risk factors and face a high risk of developing mental disorders, especially when leaving care.

**What's new?**

- Care leavers with persistent or newly developed mental disorders reported the lowest quality of life, while those without disorders showed a higher quality of life.
- Internalizing disorders in adolescence predicted poorer quality of life in adulthood, while adult internalizing and externalizing disorders were also strongly linked to reduced quality of life.

**What's relevant?**

- Continuous monitoring and effective treatment during the out‐of‐home placement and across the transition into adulthood are crucial to prevent chronicity and foster healthier life outcomes.

## Supporting information


**Table S1:** STROBE statement.
**Table S2:** Grouping of mental disorders according to the HiTOP model based on the work of Seker et al. (2022).
**Table S3:** Comparison of included (*N* = 119) and nonincluded (*N* = 473) study participants regarding main characteristics (attrition analysis).
**Figure S1:** Prevalence of mental disorders in childhood/adolescence and young adulthood (*N* = 119).
**Table S4:** Comparison of JAEL care leavers and normative data of the WHOQOL‐BREF domains.
**Table S5:** Correlation coefficients between quality‐of‐life domains and presence of any mental disorders in childhood and adolescence (*N* = 119).
**Table S6:** Correlation coefficients between quality‐of‐life domains and presence of any mental disorders in young adulthood (*N* = 119).
**Table S7:** Living situation after exiting residential care (*N* = 119).
**Table S8:** Associations between adolescent mental disorders and overall quality of life in young adulthood with psychotherapeutic treatment as covariate.

## Data Availability

The data that support the findings of this study are available on request from the corresponding author. The data are not publicly available due to privacy or ethical restrictions.
