# Supplementary material for: Mental disorder trajectories and quality of life among youth residential care leavers: a longitudinal study
Source: J Child Psychol Psychiatry. 2026 Jul 30;67(12):2099–111. doi: 10.1111/jcpp.70210 (PMC13745784; doi:10.1111/jcpp.70210)
Supplement: Supplementary file 1 [file JCPP-67-2099-s001.docx]

**Mental Disorder Trajectories and Quality of Life Among Youth Residential Care Leavers: A Longitudinal Study**

**Supporting Information**

**Summary**

[Table S1: STROBE Statement	2](#_Toc235541137)

[Appendix S1: Detailed explanation of the HiTOP model	5](#_Toc235541138)

[Table S2: Grouping of Mental Disorders According to the HiTOP model based on the work of Seker et al. (2022)	6](#_Toc235541139)

[Table S3: Comparison of included (N = 119) and non-included (N = 473) study participants regarding main characteristics (attrition analysis)	7](#_Toc235541140)

[Figure S1: Prevalence of mental disorders in childhood / adolescence and young adulthood (*N* = 119)	7](#_Toc235541141)

[Table S4: Comparison of JAEL care leavers and normative data of the WHOQOL-BREF domains	8](#_Toc235541142)

[Table S5: Correlation coefficients between quality of life domains and presence of any mental disorders in childhood and adolescence (*N* = 119)	8](#_Toc235541143)

[Table S6: Correlation coefficients between quality of life domains and presence of any mental disorders in young adulthood (*N* = 119)	8](#_Toc235541144)

[Table S7: Living situation after exiting residential care (N =119)	10](#_Toc235541145)

[Table S8: Associations between adolescent mental disorders and overall quality of life in young adulthood with psychotherapeutic treatment as covariate	11](#_Toc235541146)

# ****Table S1: STROBE Statement****

|  | Item No | Recommendation | Page No |
| --- | --- | --- | --- |
| **Title and abstract** | 1 | (*a*) Indicate the study’s design with a commonly used term in the title or the abstract | 1,2 |
|  |  | (*b*) Provide in the abstract an informative and balanced summary of what was done and what was found | 2 |
| Introduction | | | |
| Background/rationale | 2 | Explain the scientific background and rationale for the investigation being reported | 3-6 |
| Objectives | 3 | State specific objectives, including any prespecified hypotheses | 6 |
| Methods | | | |
| Study design | 4 | Present key elements of study design early in the paper | 7 |
| Setting | 5 | Describe the setting, locations, and relevant dates, including periods of recruitment, exposure, follow-up, and data collection | 7 |
| Participants | 6 | (*a*) Give the eligibility criteria, and the sources and methods of selection of participants. Describe methods of follow-up | 7 |
|  |  | (*b*) For matched studies, give matching criteria and number of exposed and unexposed | Not applicable |
| Variables | 7 | Clearly define all outcomes, exposures, predictors, potential confounders, and effect modifiers. Give diagnostic criteria, if applicable | 8-9 |
| Data sources/ measurement | 8* | For each variable of interest, give sources of data and details of methods of assessment (measurement). Describe comparability of assessment methods if there is more than one group. | 8-9 |
| Bias | 9 | Describe any efforts to address potential sources of bias | Not applicable |
| Study size | 10 | Explain how the study size was arrived at | Supplements |
| Quantitative variables | 11 | Explain how quantitative variables were handled in the analyses. If applicable, describe which groupings were chosen and why. | 8-9 |

**STROBE Statement (continued)**

| Statistical methods | 12 | (*a*) Describe all statistical methods, including those used to control for confounding | 9-10, supplements |
| --- | --- | --- | --- |
|  |  | (*b*) Describe any methods used to examine subgroups and interactions | Not applicable |
|  |  | (*c*) Explain how missing data were addressed | 9-10 |
|  |  | (*d*) If applicable, explain how loss to follow-up was addressed | 9-10 |
|  |  | (*e*) Describe any sensitivity analyses | Supplements |
| **Results** | | | |
| Participants | 13* | (a) Report numbers of individuals at each stage of study—eg numbers potentially eligible, examined for eligibility, confirmed eligible, included in the study, completing follow-up, and analysed | Supplements |
|  |  | (b) Give reasons for non-participation at each stage | Supplements |
|  |  | (c) Consider use of a flow diagram | Supplements |
| Descriptive data | 14* | (a) Give characteristics of study participants (eg demographic, clinical, social) and information on exposures and potential confounders | 10 |
|  |  | (b) Indicate number of participants with missing data for each variable of interest | Supplements |
|  |  | (c) Summarise follow-up time (eg, average and total amount) | 10 |
| Outcome data | 15* | Report numbers of outcome events or summary measures over time | Not applicable |

Checklist of items that should be included in reports of *cohort studies*

| Main results | 16 | (*a*) Give unadjusted estimates and, if applicable, confounder-adjusted estimates and their precision (eg, 95% confidence interval). Make clear which confounders were adjusted for and why they were included | 14-17 |
| --- | --- | --- | --- |
|  |  | (*b*) Report category boundaries when continuous variables were categorized | Not applicable |
|  |  | (*c*) If relevant, consider translating estimates of relative risk into absolute risk for a meaningful time period | Not applicable |
| Other analyses | 17 | Report other analyses done—eg analyses of subgroups and interactions, and sensitivity analyses | Supplements |
| Discussion | | | |
| Key results | 18 | Summarise key results with reference to study objectives | 17 |
| Limitations | 19 | Discuss limitations of the study, taking into account sources of potential bias or imprecision. Discuss both direction and magnitude of any potential bias | 20-21 |
| Interpretation | 20 | Give a cautious overall interpretation of results considering objectives, limitations, multiplicity of analyses, results from similar studies, and other relevant evidence | 18-20 |
| Generalisability | 21 | Discuss the generalisability (external validity) of the study results | 21 |
| Other information | | | |
| Funding | 22 | Give the source of funding and the role of the funders for the present study and, if applicable, for the original study on which the present article is based | 23 |

# Appendix S1: Detailed explanation of the HiTOP model

HiTOP (Hierarchical Taxonomy of Psychopathology) is a dimensional model that organizes mental disorders based on empirical patterns of symptom co-occurrence. HiTOP organizes disorders hierarchically, instead of treating them as separate categories.

There are seven subfactors which organize different diagnoses of mental disorders or syndromes: sexual problems (such as low desire and difficulties with arousal), eating pathology (such as anorexia nervosa, bulimia nervosa, and binge eating disorder), fear (such as social and specific phobias, OCD), distress (such as PTSD, major depressive disorder), mania (such as bipolar disorder I and II), substance abuse (such as substance abuse related disorders), antisocial behavior (such as conduct disorder and ADHD) are grouped into broader spectra:

- **Internalizing**: sexual problems, eating pathology, fear, distress, and mania
- **Thought Disorder**: mania and schizophrenia spectrum disorders and personality disorders
- **Detachment**: schizoid, avoidant, dependent, and histrionic personality disorder
- **Externalizing:** substance abuse and antisocial behavior

These spectra combine at the top level into the *p*-factor, representing overall vulnerability to psychopathology. The key strengths of HiTOP are its dimensional, symptom-based, and personality-informed approach, offering a more accurate and flexible alternative to traditional diagnostic systems (for more information consult: <https://renaissance.stonybrookmedicine.edu/HITOP/AboutHiTOP>).

**References**

Kotov R, Cicero DC, Conway CC, et al. The Hierarchical Taxonomy of Psychopathology (HiTOP) in psychiatric practice and research. *Psychol Med*. 2022;52(9):1666-1678. doi:10.1017/S0033291722001301

# Table S2: Grouping of Mental Disorders According to the HiTOP model based on the work of Seker et al. (2022)

|  | Grouping of disorders according to ICD-10 |
| --- | --- |
| Disorder group |  |
| Fear disorders | F40–F42, F93 |
| Distress disorders | F32–F34, F38–F38, F41, F43, F51, F93.3, F93.8, F60.31 |
| Mania disorders | F30–F31, F32.3 |
| Eating disorders | F50.1, F50.3 |
| Substance abuse | F10–F25 |
| Antisocial behavior disorders | F60.2, F60.3, F60.4, F60.81, F60.0 F63.2, F90–F92, F94 |
| Disorder spectrum |  |
| Thought disorders | F60.0, F60.1, F32.3 |
| Internalizing disorders | F32–F34, F38–F38, F41, F43, F40–F42, F50.1, F50.3, F51, F60.31, F93.3, F93.8, F93 |
| Externalizing disorders | F10–F25, F60.2, F60.3, F60.4, F60.81, F60.0, F63.2, F90–F92, F94 |
| Detachment disorders | F60.6, F60.7, F60.4, F60.1 |
| Any mental disorder | F10–F25, F32–F34, F38–F38, F41, F43, F40–F42, F50.1, F50.3, F51, F60.31, F93.3, F93.8, F93, F60.2, F60.3, F60.4, F60.81, F60.0, F60.1, F60.4, F60.6, F60.7, F63.2, F90–F92, F94 |

*Note.* ICD-10 = *International Classification of Diseases 10th Revision.* Eating disorders are not included as a diagnosis in the Structured Clinical Interview for DSM5 Disorders (SCID-5) and were thus not assessed in adulthood.

**References**

Seker, S., Boonmann, C., d’Huart, D., Bürgin, D., Schmeck, K., Jenkel, N., Steppan, M., Grob, A., Forsman, H., Fegert, J. M., & Schmid, M. (2022). Mental disorders into adulthood among adolescents placed in residential care: A prospective 10-year follow-up study. European Psychiatry, 65(1), e40. https://doi.org/10.1192/j.eurpsy.2022.30

# Table S3: Comparison of included (N = 119) and non-included (N = 473) study participants regarding main characteristics (attrition analysis)

|  | **Included** | **Non-included** | **Test-Statistic** | **Significance** |
| --- | --- | --- | --- | --- |
|  | N (%) | N (%) |  |  |
| Age at baseline (years), *M* [*SD*] | 15.1 [2.2] | 16.1 [3.1] | *t*(254) = -4.1 | *p* < .001 |
| Women | 42 (35.3) | 148 (31.3) | χ^2^ = 0.53, df = 1 | *p* = .47 |
| Swiss nationality | 94 (79.0) | 366 (77.4) | χ^2^ = 0.07, df = 1 | *p* = .80 |
| Language region  German-speaking  French-speaking  Italian-speaking | 81 (68.1  16 (13.5)  22 (18.5) | 291 (74.2)  41 (10.5)  60 (15.3) | χ^2^ = 5.5, df = 2 | *p* = .07 |
| Any mental disorder at baseline (K-SADS) | 81 (68.1) | 258 (70.9) | χ^2^ = 0.22, df = 1 | *p* = .64 |
| Age at first placement (years), *M* [*SD*] | 11.2 [4.6] | 12.0 [4.8] | *t*(192) = -1.14 | *p* = .26 |
| Number of placements, *M* [*SD*] | 3.5 [3.4] | 4.0 [3.9] | *t*(180) = -1.09 | *p* = .28 |
| Approximate time between beginning of first and end of last placement (years), *M* [*SD*] | 6.5 [4.6] | 7.5 [5.4] | *t*(177) = -1.37 | *p* = .17 |

*Notes***.** N=number of participants, *M* = mean, *SD* = standard deviation, K-SADS = Kiddie Schedule for Affective Disorder and Schizophrenia – Present and Lifetime Version. Due to missing data, the number of non-included participants does not add up to N = 473 in all variables included in this table.

# Figure S1: Prevalence of mental disorders in childhood / adolescence and young adulthood (*N* = 119)

# Table S4: Comparison of JAEL care leavers and normative data of the WHOQOL-BREF domains

| QoL-domains | JAEL sample  *N* = 119  *M* (*SD*) | Normative data  *N* = 394  *M* (*SD*) | *p*-value | Effect size |
| --- | --- | --- | --- | --- |
| Physical | 73.4 (17.2) | 84.1 (14.0) | <.001 | 0.62 |
| Psychological | 62.7 (20.1) | 77.6 (15.0) | <.001 | 0.74 |
| Social | 68.1 (21.7) | 74.8 (18.6) | <.001 | 0.31 |
| Environmental | 68.7 (16.2) | 71.5 (13.5) | 0.06 | 0.17 |
| Total | 68.2 (15.7) | 72.3 (15.7) | <.001 | 0.32 |

# Table S5: Correlation coefficients between quality of life domains and presence of any mental disorders in childhood and adolescence (*N* = 119)

|  | Externalizing | Internalizing | Thought | Detachment |
| --- | --- | --- | --- | --- |
| Physical QoL | 0.00 | -0.23** | -0.04 | -0.11 |
| Psychological QoL | 0.06 | -0.25** | -0.09 | -0.00 |
| Social QoL | 0.06 | -0.15 | -0.01 | 0.05 |
| Environmental QoL | -0.00 | -0.20* | -0.13 | 0.05 |

*Note*. ****p* < 0.001, ***p* < 0.01, **p* < 0.05. Values represent point-biserial correlations between mental disorders at baseline (HiTOP Spectra) and QoL domains.

# Table S6: Correlation coefficients between quality of life domains and presence of any mental disorders in young adulthood (*N* = 119)

|  | Externalizing | Internalizing | Thought | Detachment |
| --- | --- | --- | --- | --- |
| Physical QoL | -0.21* | -0.43*** | -0.17 | -0.18 |
| Psychological QoL | -0.21* | -0.36*** | -0.09 | -0.13 |
| Social QoL | -0.06 | -0.19*** | -0.14 | -0.19* |
| Environmental QoL | -0.15 | -0.36*** | -0.11 | -0.07 |

*Note*. ****p* < 0.001, ***p* < 0.01, **p* < 0.05. Values represent point-biserial correlations between mental disorders at follow-up (HiTOP Spectra) and QoL domains.

# Table S7: Living situation after exiting residential care (*N* =119)

| **Living situation after exiting residential care** | ***n* (%)** |
| --- | --- |
| Living with partner | 47 (39.5) |
| Living alone | 39 (32.8) |
| Shared living | 13 (10.9) |
| Living with parents | 12 (10.1) |
| Residential care | 5 (4.2) |
| Foster family | 1 (0.8) |
| Relatives | 1 (0.8) |
| Friends | 1 (0.8) |

# Table S8: Associations between adolescent mental disorders and overall quality of life in young adulthood with psychotherapeutic treatment as covariate

| Dependent variable | Independent variable | Stand. Beta | *SE* | *p* value | 95% CI | *f*^2^ |
| --- | --- | --- | --- | --- | --- | --- |
| **Overall QoL** | Any mental disorder | −0.15 | 0.185 | ns | -0.51, 0.21 | 0.19 |
|  | Gender | −0.42 | 0.183 | <.05 | -0.78, -0.06 |  |
|  | Age | 0.11 | 0.089 | ns | -0.05, 0.28 |  |
|  | Psychotherapeutic treatment | -0.81 | 0.21 | <.001 | -1.23, -0.39 |  |
| **Overall QoL** | Externalizing disorders | 0.06 | 0.174 | ns | -0.29, 0.40 | 0.18 |
|  | Gender | -0.43 | 0.183 | <.05 | -0.79, -0.06 |  |
|  | Age | 0.09 | 0.090 | ns | -0.84, 0.27 |  |
|  | Psychotherapeutic treatment | -0.82 | 0.210 | <.001 | -1.24, -0.41 |  |
| **Overall QoL** | Internalizing disorders | −0.32 | 0.199 | ns | -0.72, 0.07 | 0.21 |
|  | Gender | −0.33 | 0.292 | ns | -0.71, 0.07 |  |
|  | Age | 0.08 | 0.089 | ns | -0.09, 0.25 |  |
|  | Psychotherapeutic treatment | -0.77 | 0.211 | <.001 | -1.18, -0.35 |  |
| **Overall QoL** | Detachment disorders | 0.07 | 0.381 | ns | -0.69, 0.82 | 0.23 |
|  | Gender | -0.42 | 0.182 | <.05 | -0.78, -0.06 |  |
|  | Age | 0.27 | 0.119 | <.05 | 0.03, 0.50 |  |
|  | Psychotherapeutic treatment | -0.83 | 0.204 | <.001 | -1.24, -0.43 |  |
| **Overall QoL** | Thought disorders | -0.22 | 0.500 | ns | -1.21, 0.77 | 0.19 |
|  | Gender | -0.44 | 0.186 | <.05 | -0.81, -0.07 |  |
|  | Age | 0.11 | 0.090 | ns | -0.07, 0.28 |  |
|  | Psychotherapeutic treatment | -0.80 | 0.216 | <.001 | -1.23, -0.38 |  |

*Note*. Mental disorders were assessed with the Kiddie Schedule for Affective Disorder and Schizophrenia—Present and Lifetime Version (K-SADS-PL) and the Structured Clinical Interview for DSM-IV-TR Axis II Personality Disorders (SCID-II) and classified based on the HiTOP spectrum categories. *SE* = standard error; CI = confidence interval; ns = not significant (*p* > .05); *f*^2^ = effect size (values of 0.02, 0.15, and 0.35 indicate small, medium, and large effects, respectively).
